# A Drug Safety Concept (I) to Avoid Polypharmacy Risks in Transplantation by Individual Pharmacotherapy Management in Therapeutic Drug Monitoring of Immunosuppressants

**DOI:** 10.3390/pharmaceutics15092300

**Published:** 2023-09-10

**Authors:** Ursula Wolf

**Affiliations:** Pharmacotherapy Management, University Hospital Halle (Saale), Martin Luther University Halle-Wittenberg, 06120 Halle (Saale), Germany; ursula.wolf@uk-halle.de

**Keywords:** transplantation, therapeutic drug monitoring (TDM), immunosuppression, calcineurin inhibitors, electronic patient record, electronic health record (EHR), medication review, polypharmacy, adverse drug reactions (ADRs), drug-drug interactions (DDIs), drug elimination capacities, graft safety, patient safety, drug safety

## Abstract

For several, also vital medications, such as immunosuppressants in solid organ and hematopoietic stem cell transplantation, therapeutic drug monitoring (TDM) remains the only strategy for fine-tuning the dosage to the individual patient. Especially in severe clinical complications, the intraindividual condition of the patient changes abruptly, and in addition, drug-drug interactions (DDIs) can significantly impact exposure, due to concomitant medication alterations. Therefore, a single TDM value can hardly be the sole basis for optimal timely dose adjustment. Moreover, every intraindividually varying situation that affects the drug exposure needs synoptic consideration for the earliest adjustment. To place the TDM value in the context of the patient’s most detailed current condition and concomitant medications, the Individual Pharmacotherapy Management (IPM) was implemented in the posttransplant TDM of calcineurin inhibitors assessed by the in-house laboratory. The first strategic pillar are the defined patient scores from the electronic patient record. In this synopsis, the Summaries of Product Characteristics (SmPCs) of each drug from the updated medication list are reconciled for contraindication, dosing, adverse drug reactions (ADRs), and DDIs, accounting for defined medication scores as a second pillar. In parallel, IPM documents the resulting review of each TDM value chronologically in a separate electronic Excel file throughout each patient’s transplant course. This longitudinal overview provides a further source of information at a glance. Thus, the applied two-arm concept of TDM and IPM ensures an individually tailored immunosuppression in the severely susceptible early phase of transplantation through digital interdisciplinary networking, with instructive and educative recommendations to the attending physicians in real-time. This concept of contextualizing a TDM value to the precise patient’s condition and comedication was established at Halle University Hospital to ensure patient, graft, and drug safety.

## 1. Introduction

Therapeutic drug monitoring (TDM) is particularly important for drugs that are known to have a narrow therapeutic window, with serious drug and adverse effects outside this range. The real-world risks become all the more critical for those that additionally exhibit a high potential for drug-drug interactions (DDIs). This applies, for example, to various antiepileptic drugs, chemotherapeutics, antimicrobials, and immunosuppressants. In transplantation, the standard immunosuppressants’ TDM is essential from both points of view. For the calcineurin inhibitors, cyclosporine A (CsA) and tacrolimus (TAC), as well as the mammalian targets of rapamycin inhibitors (mTORIs), everolimus and sirolimus, there is a small therapeutic range, as well as the enhanced DDI risks from unavoidable polypharmacy, e.g., from concomitant various antimicrobials in the acute, most vulnerable posttransplant phase in particular.

Furthermore, the patient’s varying clinical conditions with impact on liver function determine the TDM values of CNIs. Knowing the correct exposure and inter- and intraindividually varying elimination capacities for the immunosuppressant in the individual transplant patient with all of his/her comorbidities and concomitant medications ensures optimized maneuvering through this high-risk posttransplant phase, which remains a most important determinant of patient and transplant outcomes. Throughout the last decades of transplantation medicine, the impact of adequate immunosuppression has been studied to optimize the acute and long-term results in organ transplantation [1,2,3,4] and allogeneic hematopoietic stem cell transplantation (HSCT) [5]. The intraindividual trough-level variability following kidney transplantation even predicts long-term allograft survival [3,4].

Accordingly, not only the results of TDM measures but also the often abrupt changes in concomitant medication or reduction in drug degradation/excretion capacities require our maximum attention. This is to adjust the dose as early as possible and to avoid the inappropriate exposure of immunosuppressants from the very beginning in transplantation conditions.

To address these issues simultaneously and to manage all challenges as safely as possible are the aims of an accordingly designed and, for the last 9 years, established individual TDM in conjuncture with an effective Individual Pharmacotherapy Management (IPM).

This is the first of a two-part publication. It is to introduce a thoroughly approved and reproducible concept. In a second article, the most relevant findings from this real-world experience will be presented to further specify corresponding clinical practice recommendations with respect to the contemporary unavoidable acute transient and long-term polypharmacy in transplantation.

## 2. Concept and Implementation of a Digital Interdisciplinary Networking Strategy

### 2.1. Presentation of the Designed IPM-TDM Concept

**Figure 1:**
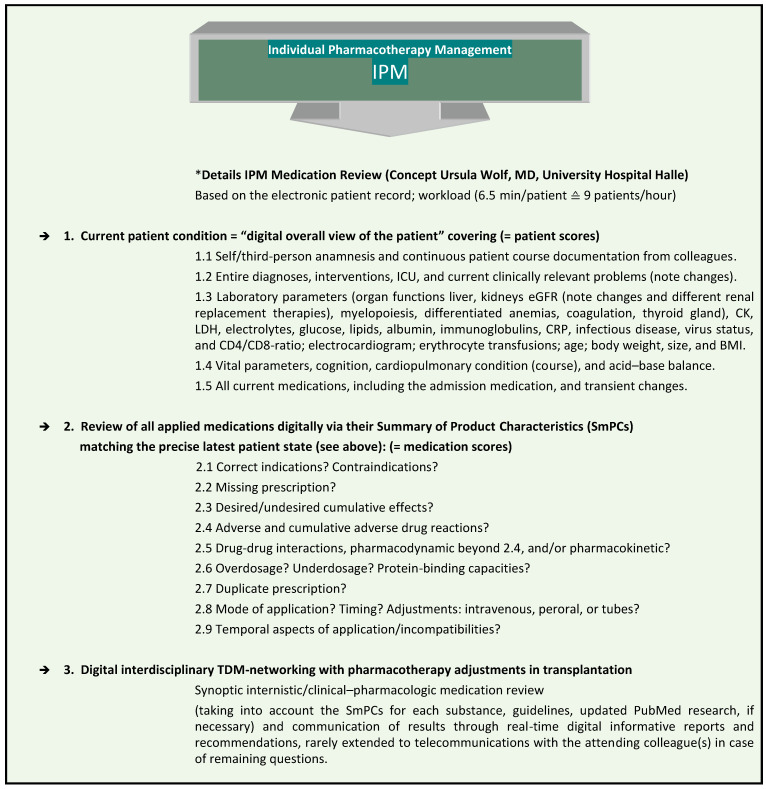
Comprehensive, reproducible IPM based on the electronic patient record. * IPM (applied patient and medication scores) based on the electronic hospital patient records at Halle University Hospital. This was conceptualized, implemented, and practiced by Wolf, MD, Head of Pharmacotherapy Management Department, specialist in internal medicine, with expertise in clinical pharmacology and transplantation, and performed > 59,700 individual IPM medication reviews.

**Figure 2:**
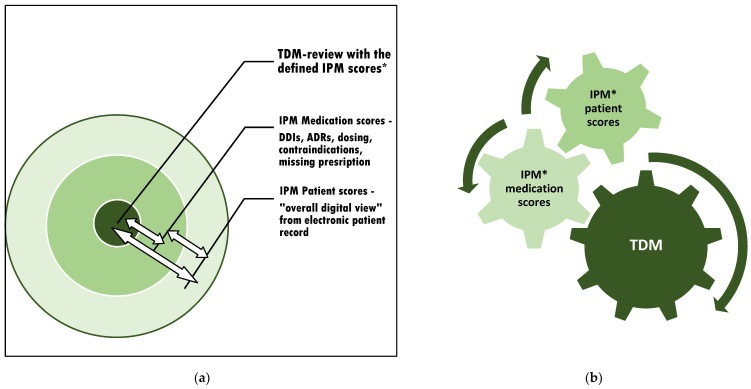
The context of appropriate TDM assessment and subsequent adjustment: (**a**) The individual circuits affect each other. Therefore, interference monitoring is required. (**b**) Optimization of the TDM value by continuous synoptic contribution and adaptation to confounding risks from varying current patient conditions and altering comedications through simultaneous IPM in real-time. * IPM scores: see Figure 1.

At Halle University Hospital (UKH), the IPM procedure has been established for nine years (Figure 1), and has been approved to be significantly associated with the successful prevention of risks from polypharmacy [6,7,8,9]. It is performed as a synopsis of internal medicine and clinical pharmacology, according to the clinical education of the responsible IPM physician, who also has six years of clinical expertise in transplantation. The IPM is combined with the individual trough level TDM of immunosuppressants in patients undergoing solid organ or allogeneic hematopoietic stem cell transplantation (HSCT) (Figure 2). It provides continuous interdisciplinary networking based on the clinical electronic health record in each patient from the very onset of transplantation. The reproducible IPM protocol refers to the most accurate current clinical condition of the patient with respect to his/her organ functions and vital parameters. In order to match the prescribed medications to the patient’s degradation and elimination capacities, taking into account the real-time concurrently manifested pharmacokinetic DDIs, the entire medication list is analyzed on the basis of the Summaries of Product Characteristics (SmPCs) for ADRs, including pharmacodynamic DDIs, contraindications, warnings, and dosage. Additional tools are used in complicated situations, such as continuous renal replacement therapy (CRRT) [10,11,12] and for further interaction checks in case of open questions [13,14] and PubMed research.

This procedure respects the most detailed real-time patient condition while contributing to each circuit that affects the intraindividual exposure of the monitored drug (Figure 2a). The method is applicable to all kinds of TDM-managed medications to accurately relate the TDM value to the patient’s overall clinical situation. For the most precise consecutive TDM dose adjustment, it is important to refer to the IPM-defined patient and medication scores simultaneously (Figure 2b).

**Figure 3:**
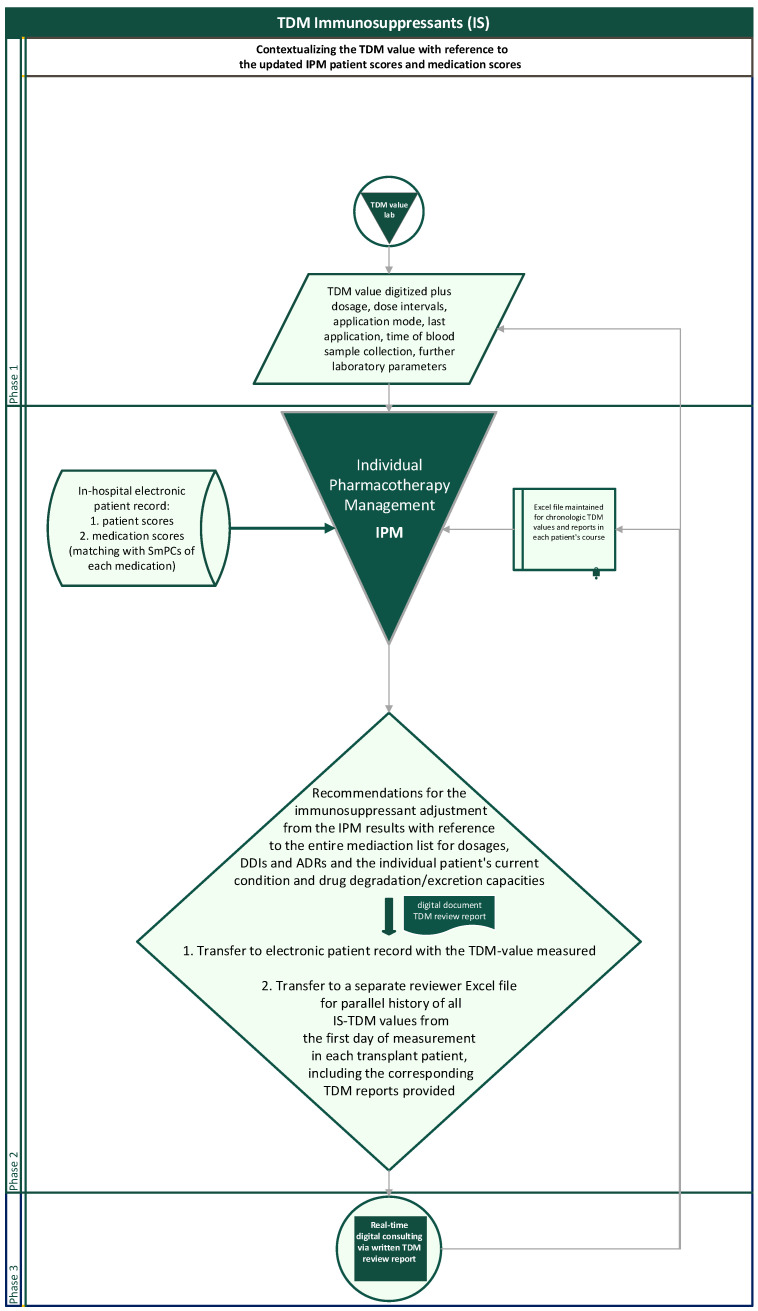
Flowchart of the routinely contextualized TDM of immunosuppressants with the IPM concept at Halle University Hospital.

The flow chart illustrates the entirely reproducible method applied (Figure 3). For interpreting concentration measurements, the dosage and dosing intervals, the sampling time in relation to application time, the sampling modus with risks from contamination in case of catheter blood samples (pre-analytic errors), and the questionable steady state in case of therapy initiation, dose adjustments, new DDIs, or reduced metabolism depending upon the drug’s half-life are the first steps taken into account. With regard to the desired TDM target range, which is particularly dependent upon the transplantation phase and the individual course, a necessarily transient, abruptly changing target range for the desired TDM value, e.g., due to rejections or CMV infections, or a further intensified alternative immunosuppressive therapy must always be considered.

In a separate Excel document, the IPM-TDM consultant additionally creates and maintains a parallel longitudinal Excel file of the entire engraftment history of TDM values and corresponding reports for each patient to provide an intraindividual chronologic overview at a glance when entering a new TDM reading of the patient (Figure 3). The history overview of the reports is also available for any attending physician in the electronic patient record with the chronologic TDM values.

### 2.2. Examples of Implementation with Digital Interdisciplinary Networking Reports

Resulting actual examples of IPM-TDM reports from the implementation of this concept, always provided digitally with the name of the IPM reviewer and the telephone number for very rare requests, reflect the real-world clinical environment of a TDM value to be considered for graft and patient safety from the very beginning. The intention is to report the IPM-TDM findings as concisely as possible.
**Patient 10 days post-HSCT**, “CsA trough level in the upper therapeutic range of the acute HSCT phase. Fluconazole, amlodipine, and carvedilol increase the level. In particular, fluconazole reduces fentanyl degradation with an urgent need for fentanyl re-evaluation, especially in the case of withdrawal of metamizole as current cytochrome P450 isozyme 3A4 (CYP3A4)-inducer. Metamizole also lowers CsA levels. Cave: Coxib in the PRN medication is considered critical in combination with CsA, risk of additive nephrotoxicity. In addition, always keep in mind the myelosuppressive potential of mirtazapine in the context of HSCT. Increased risk of bleeding with duloxetine, cave with current thrombocytopenia. Manifest hypomagnesemia under CsA. Wolf, tel. no.” **Patient 4 days post-HSCT**, “Incorrect dose information? Exclude sampling error. TAC trough level inadequately increased and above therapeutic range of the acute HSCT phase with risks for kidneys (see declining e GFR) and infections. Voriconazole increases TAC-level, as do yesterday’s red blood cell transfusions. Aprepitant starting tomorrow will increase CsA exposure further. Cave: Enhanced exposure of loperamide under tacrolimus and voriconazole (cumulative risk of long QTc). Especially critical with currently manifest hypomagnesemia. Wolf, tel. no.” **Patient 10 years after lung transplantation hospitalized in the orthopedic department for operative intervention**, “TAC trough level in the therapeutic range of long-term therapy under concomitant inhibitors of metabolism itraconazole and amlodipine and on the other side the CYP3A4 inducer metamizole. Cave: If metamizole is discontinued, TAC levels will rise, requiring close monitoring and timely dose adjustment. Maintain serum potassium (K) and magnesium (Mg) high normal with current cumulative risk of QTc elongation. The actual TAC target range depends on the primary clinical problem, (CMV diagnostics?—PCR? and/or CD4/CD8-ratio inversed?) and the course of the graft. Avoid combination of coxib and TAC with risk of cumulative nephrotoxicity. Wolf, tel. no.” **Patient 6 months post-HSCT**, “CsA trough level despite low dosage inadequate and again within a therapeutic range of an acute therapy phase with risk of infections and nephrotoxicity. Exclude sampling error. Current target range always according to foreground clinical risk and HSCT course as well as further immunosuppression. The acute discrete liver dysfunction, depending on varying extent, leads to impaired metabolism of CsA, among others. Furthermore, ponatinib increases CsA exposure and requires dose adjustment of CsA and follow-up as well as monitoring of liver function. Additional findings: Differential diagnosis CMV? Previous folic acid deficiency now compensated? Wolf, tel. no.” **Patient 3 years after kidney transplantation**, “TAC level delayed by 2 h below measurable range at minimum daily dose and single dose. However, transient cortisone bridging has been introduced. Current TAC target range depends on the acute priority clinical problem. Lercanidipine increases TAC exposure. Recent manifestations of hyponatraemia, folic acid deficiency, hypogammaglobulinemia (potential ADR of MMF). Intermittent serum Mg monitoring is recommended. Very critical is the combination of 40 mg simvastatin with lercanidipine, which leads to a considerably higher statin exposure with renal risks, obviously used in long-term therapy with CKD of the kidney transplant. Wolf, tel. no.” **Patient 8 weeks after HSCT**, “CsA trough level in the therapeutic range of a post-acute phase of HSCT. Voriconazole increases the CsA exposure. Current target range according to frontline clinic (CMV?) and HSCT course. Reversal of CD4/CD8 ratio inversion (besides PCR) after virustatic therapy? Manifest hypogammaglobulinemia after MMF as a potential ADR? Probenecid is contraindicated in patients with eGFR < 50 mL/min/1.73 m^2^ and critical in pre-existing hematopoietic disorders (see SmPC). Renal insufficiency [creatinine clearance ≤ 55 mL/min or ≥2+ proteinuria (>100 mg/dL)] is a contraindication to the use of cidofovir (see SmPC). Manifest hypomagnesemia, critical with current cumulative long-QTc risk from voriconazole, levofloxacin, etc. Maintain high normal serum K and Mg in this high-risk constellation if unavoidable. Wolf, tel. no.” 

### 2.3. Comparative Aspects to Standard Procedure

The standard TDM, which usually only gives the measured value in relation to a defined therapeutic range, although this may even demand adjustments to the patient’s and graft’s courses as a patient’s concomitant disease progresses, is without any context. In contrast, the applied concept addresses the entire acute patient condition and his/her current overall medication. In particular, the focus is on the predominant arising clinical problems that may require correspondingly necessary changes in the therapeutic target range, such as severe CMV infectious disease on the one hand or rejection on the other. The potentially nephrotoxic effects of the CNIs themselves, especially with excessive blood levels and increased exposure, are contextualized with the overall additional risk from the patient’s concomitant medication. This is for patient and graft safety, preventing harm by adjusting each medication as early as possible. Accordingly, it enables emerging risks to be identified in advance and targeted countermeasures to be taken.

The entire IPM-TDM process takes an average of only 6.5 min per patient with a daily routine and a comprehensive electronic medical record. It enables seamless, digital, and real-time interdisciplinary networking and is applicable to any TDM drug dosing. The resulting recommendations for the attending colleagues are communicated digitally immediately and, in rare cases only, also telemedically for remaining open questions. In addition to the predominant aspect of gathering all patient information from the most comprehensive digital overview through the patient’s electronic health record, this interdisciplinary digital networking strategy saves enormous amounts of time. Compared to on-site consultations, which may take more than half an hour, including time to get to each patient, this tight digital networking makes it possible to follow transplant patients around the UKH in all the different departments on a daily basis, which would otherwise be impossible to achieve, due to time and staffing constraints.

## 3. Discussion

Reducing toxicity while maintaining anti-rejective efficacy impacts the outcome of the patient and graft in solid organ and HSCT and remains one of the most important challenges in immunosuppression posttransplant. The applied concept aims to optimize the drug dosage, excluding any risks of over- and underdosing (1) in the individual patient and transplant condition (2) at the earliest stage. It has been implemented routinely for TDM measurements of the calcineurin inhibitors CsA and TAC at the UKH in solid organ and HSCT patients and patients treated for immunologic diseases. The presented, approved TDM method can be applied analogously to all kinds of other drugs. The predominant and essential focus of this drug safety concept is the current and most concise overall patient condition and concomitant medication list to be contextualized with the TDM value. Because this contributes to the interindividual and intraindividual variations in exposure within equal doses applied to different patients or to the same patient in their or in his/her clinical situations and concurrent diseases, complications, and comedications changing. Polypharmacy with increasing numbers of drugs to treat the comorbidities of the transplant patients requires an accurate prescribing of each in terms of dosing, pharmacokinetic and pharmacodynamic DDIs, and ADRs, excluding contraindication, especially with regard to the patient’s clinical transplant condition, the transplant itself, and the individual extent of the concomitant immunosuppressive therapy. Currently, there is no additional routine analysis at the UKH for further intraindividual impacts of the genotype in terms of metabolic polymorphism, such as in the metabolizing enzymes cytochrome P450 (CYP) 3A4 and CYP3A5 and the multidrug efflux pump P-glycoprotein, encoded by MDR-1 [15,16,17]. Maintaining a parallel chronologic patient TDM file additionally provides easy and reliable detection of implausible individual TDM values, including those due to pre-analytic errors, such as drug contamination from intravenous administration, or inadequate time interval between blood collection and the last dosing, non-adherence, or severe absorption disorders, including inflammation- or drug-induced dysphagia [18]. The effect of timely antecedent erythrocyte transfusions also needs to be considered [19].

The partly life-saving consequences of TDM in transplantation continue to pose a challenge to achieving the most precisely tailored individual dose. For this purpose, professional work groups involved in different analytic and evaluating phases of the TDM procedures have regularly aimed at providing guidelines for improved immunosuppression, such as the report of the European consensus conference in 2009 on opportunities to optimize tacrolimus therapy in solid organ transplantation [20], the Second Consensus Report Therapeutic Drug Monitoring of Tacrolimus-Personalized Therapy, recommendations of the International Association of Therapeutic Drug Monitoring and Clinical Toxicology (IATDMCT) on the therapeutic drug monitoring of tacrolimus [21]. In the search for optimal target TDM levels, the TRAM study found no difference In CsA-induced toxicity with the area under the curve (AUC)-based TDM versus the trough-based TDM, although target values were achieved earlier and maintained more consistently in the AUC group [22]. In addition, inter- and intrapatient variabilities in pharmacokinetics (PK) have been quantified for population PK models to be used to predict the optimal dose of a drug in an individual patient. The IPM does not apply model-informed precision dosing (MIPD). As from kidney and liver transplant recipients, it has been stated that significant DDIs and the different formulations of tacrolimus need to be taken into account for any future tacrolimus population PK model development [23]. They have not been widely adopted in clinical transplantation practice, although there is some published evidence of the beneficial effects of MIPD [24,25,26,27]. In particular, transplant patients and patients on chemotherapeutic regimens prone to polypharmacy are susceptible to suboptimal medical therapy, and refined dosing is essential to improve drug treatment outcomes. In this context, each step forward must be respected, and the future direction should be to bring them together to achieve the desired optimal efficacy with minimal toxicity in all the patients with their individual age, organ conditions, polypharmacy risks, drug-relevant genetics, and even inter- and intravariable pharmacodynamic drug effects.

The TDM concept meets all the desired aspects outlined, stating that “TDM today is an individualized intervention that aims to achieve the best benefit/risk ratio, in the context of the patient and his/her entire environment” [28]. The electronic health record makes this comprehensive view of the patient and his/her environment possible. The weaknesses of merely interpreting TDM values in terms of therapeutic range include the lack of individualization, since it does not meet the individual risk/benefit profile [29]. For example, a more individualized pharmacokinetic dosing of vancomycin in a critically ill patient contributed to a faster achievement of the target value without increased nephrotoxicity [30]. Recently, the patient C-reactive protein (CRP) was identified as a significant covariate affecting the pharmacokinetics of CsA, indicating that a higher daily dose is required to achieve therapeutic trough concentrations at high CRP levels [31]. Since each patient has unique characteristics, immunosuppression has to be questioned to follow a uniform regimen [32]. For example, in the older transplant patients, acute rejection episodes are less frequent, but they are more likely to die from infectious and cardiovascular risks than younger patients [33]. The IPM-TDM concept takes into account an extensive spectrum of parameters of the individual patient in his/her precise current clinical condition and the overall comedication, as well as the dosing history, enabling the prediction of exposure and strategic intervention. Additionally, according to Holford and colleagues, this means overcoming the practical challenge of “entering of essential information as a dosing tool” for target concentration intervention (TCI) instead of a mere TDM, which is associated with less individualization of dosing and inferior outcomes. They concluded that “in the digital age TDM should be abandoned and replaced by TCI” [29].

In addition, the immediate consultation through the digitally provided TDM review report is likely to also have an educative impact on the attending physicians. In particular, various medical disciplines are not as familiar with immunosuppressive regimens in transplantation when they necessarily care for the transplant patient within a completely unrelated medical specialty for other interventions and pharmacotherapies. This can lead to high-risk conditions for the graft and the patients themselves. The real-time link to the TDM review report guarantees essential guidance for individualizing the transplant patient’s immunosuppression during any concerning and critical clinical conditions, which often involve a major change or increase in additional, potentially interacting drugs. In this context, the informative and educative component of the TDM review report is indispensable and mandatory, and it is highly desired and appreciated by the physicians in charge. The digital concept, based on the most comprehensive view of the patient possible, provided by the entire electronic medical record, furthermore constitutes an alternative, effective paradigm to traditional time-consuming on-site consultations. It can be applied analogously to the TDM of any drug and is an important step forward in addressing the global challenge of digitized interdisciplinary healthcare for the patient and drug safety, which is even more compelling in the context of drugs with a narrow therapeutic safety index and polypharmacy and in acute and long-term graft surveillance.

## 4. Strengths and Weaknesses

IPM-based TDM assessment requires a holistic view of the patient and a comprehensive medication analysis, both of which impact the exposure of calcineurin inhibitors. The educational background of the designing and performing internist, who has advanced education in clinical pharmacology, further covers a broad range of expertise and successful engagement in improving outcomes in clinical transplantation [34,35,36,37,38,39,40,41,42,43,44,45,46,47,48,49,50,51], along with the Transplantation Society Academy Distinguished Educator qualification, all of which provide experience and the necessary professional legitimacy to relate a TDM value for an immunosuppressant to the clinical condition of the patient and to the transplant itself in a broad professional context. It enables skilled individual focus and trained placement of each TDM value in its overall environmental setting for a comprehensive assessment, alongside 21 years of daily experience in TDM for immunosuppressants in transplantation.

The additional history backup of all TDM values and reports for each transplant patient enables the rapid detection of pre-analytic errors, which may result from confounding factors such as collection time, collection mode with the risk of contamination, or steady state. All TDM methods may inhere some limitations in themselves and their practical applications. Analytical aspects regarding different assays available, the most appropriate matrix, and methods of measurement are not addressed [52]. The potential analytic confounding factors from inactive metabolites or from given defined therapeutic ranges with applied analytical test agents, known to contribute to variability of TDM targets [53], are not considered within the topical context of the presented TDM concept. At UKH, there is no routine analysis of the CYP3A4, CYP3A5, or MDR-1 genotype for additional pharmacokinetic genotype-based individualized CsA or TAC dosing. In line with its widespread use, we measure TDM trough levels in adults and in children, although young age accounts for even enhanced pharmacokinetic variability [54]. There is no further AUC-based TDM or monitoring of more sensitive drug levels 2 h after dosing for comparison [55]. We do not refer to a strict dose conversion ratio of 1:2 when switching from the twice-daily infusion to oral administration, which is suggested as the most appropriate [56]. With the exception of grapefruit juice and St. John’s wort, which should be requested by the attending physician, the TDM does not cover foods and substances related to complementary and alternative medicine (CAM), such as dietary supplements, herbs, and other manufactured ingredients, although these have been shown to be of further relevant impact, especially in outpatients, as well as in cancer therapy, with a likelihood as high as 37% for interactions in the case of CAM supplements and 29% of all patients for foods [57,58].

## 5. Conclusions and Outlook

The article presents the well-established concept of a standardized longitudinal TDM of CNIs in organ and hematopoietic stem cell transplantation in synopsis with a comprehensive Individual Pharmacotherapy Management (IPM) at the UKH. This TDM concept is not limited to measured values and dose adjustment but thoroughly evaluates the relevant impact of the individual patient and transplant course, individual organ capacities for drug degradation and/or excretion, DDIs, and single and cumulative ADRs and contraindications. The individual longitudinal follow-up of measurements contextualizing all concomitant medications, comorbidities, and the changing clinical status of the patient and complications that alter the patient’s condition and clinical situation, e.g., surgical procedures, wound healing, transient infectious diseases, myelosuppressive disorders, and daily renal and hepatic function, allows for fine-tuned and partially predictable post-transplant immunosuppressant adjustment for optimized graft, patient, and drug safety.

The IPM enables the identification of neglected or unaware and often high-risk confounders. It outlines how these, or their consequences, can be avoided in the clinical setting, even in vulnerable at-risk transplant recipients or critically ill patients on polypharmacy.

The 2019 WHO report on medication safety in polypharmacy states: “A comprehensive medication review is a multidisciplinary activity whereby the risks and benefits of each medicine are considered … It optimizes the use of medicines for each individual patient…Polypharmacy can put the patient at risk of adverse drug events and drug interactions when not used appropriately.” ([59], p. 7). The intentionally comprehensive individual TDM concept fulfills the requirements of the global challenges that the WHO continues to address, and it has been established at UKH for nine years. Its acceptance is very positive, and the interdisciplinary networking does not require any time-consuming activities for the attending physicians, as each TDM review report is digitally transmitted and presented with the laboratory-measured TDM value. The resulting expertise on the most relevant and common real-world IPM findings in TDM of calcineurin inhibitors in the context of contemporary and inevitable polypharmacy in transplantation is the subject of the second article in this series.

The presented concept is applicable to all TDM-managed medications. In particular, it can be usefully applied in transplantation medicine for the acute and long-term follow-up, in life-threatening severe immunologic diseases requiring, for example, immunosuppressive therapy with optimized dosing of CNIs or mTORIs, or in chemotherapeutic therapy with drug availability susceptible to DDIs and patient condition.

As a consequence, the substantial real-world experience gained over many years is to be transferred into a clinical software program for use in conjunction with the electronic patient record to make it available for the treating physicians in digitalized form. To my knowledge, other than clinical decision support systems [60], there is no database that is integrated with an electronic health record system to perform this IPM in the holistic elaborate manner of the concept presented, including comprehensive patient and medication scores. Although, today’s concomitant polypharmacy frequently leads to concerning medical findings. These must be taken into account as early as possible to prevent harm and to ameliorate the patient’s clinical course and long-term graft survival, still eagerly awaited in solid organ transplantation and with an organ shortage. A European cohort analysis based on the Collaborative Transplant Study demonstrated that even the five-year improvement in kidney allograft survival declined from 2000 to 2015, indicating the remaining unmet demand for innovation in this early transplant area as well [61].

## Data Availability

There were no datasets generated and analyzed for the concept presented. Anonymized IPM-TDM reports are available from the author upon request.
